# Editorial: Insights in diabetes: molecular mechanisms 2022

**DOI:** 10.3389/fendo.2023.1242759

**Published:** 2023-08-07

**Authors:** Gundu H. R. Rao

**Affiliations:** Laboratory Medicine, and Pathology, University of Minnesota, Minneapolis, MN, United States

**Keywords:** hypertension, excess weight, obesity, diabetes, vascular diseases, diabetes: molecular mechanisms

It gives me great pleasure in writing this invited editorial for the Research Topic in Frontiers in Endocrinology titled “*Insights in diabetes: molecular mechanisms 2022*.” Considering the fact that diabetic vascular disease is responsible for two- to four-fold increases in the occurrence of coronary artery disease and stroke, and two- to eight-fold increases in the risk of heart failure, I expected several articles relating to the basic cellular and molecular mechanisms and to glucose homeostasis. Of the three articles accepted for publication in this Research Topic, Hou et al. from China summarize the physiological significance of vasoactive intestinal peptide (VIP) in glucose homeostasis. The authors speculate that this peptide may act as an endogenous insulinotropic drug, and as such, may not increase hypoglycemia. The authors discuss the therapeutic potentials and possible roles of its receptor (VPAC2) agonists in type 2 diabetes. The second article by Nunez Lopez et al. from the US describes the regulation of gene expression and microRNA changes in adipose tissue and circulating extracellular vesicles (EV) in response to pioglitazone treatment in humans with type-2 diabetes. The authors speculate that the beneficial effects of pioglitazone are mediated by adipose-specific miRNA regulation and exosomal EV/trafficking. Rengachar et al. from India, using an animal model, demonstrate the protective role of a metabolite of docosahexaenoic acid, Protectin DX, against drug-induced type-1 and type-2 diabetes.

Cardiometabolic diseases, such as hypertension, excess weight, obesity, type-2 diabetes, and vascular diseases, have increased in incidence and prevalence to epidemic proportions worldwide in the last four decades (1). According to the World Health Organization, a new study from Imperial College London reports a tenfold increase in childhood and adolescent obesity in the last four decades. The study, which was published in the journal Lancet ahead of World Obesity Day, is one of the largest ever in terms of the number of participants (31.5 million aged 5 to 19 and 130 million aged 20 and older). The authors conclude that, if the trend continues, more children and adolescents will be obese than moderately or severely underweight by 2022 (2). Cardiovascular diseases rank as the number one cause of death worldwide. In 2020, 19 million deaths were attributed to CVD globally (3). We are of the opinion that early diagnosis of metabolic disease risks, and management of these risks, is the only way to reduce or reverse the trend in the increase and prevalence of cardiovascular disease. To achieve these goals, we need to gain insights into the mechanisms underlying the initiation and progression of metabolic diseases, such as hypertension, obesity, and vascular diseases. In this Research Topic, overviews are presented on glucose homeostasis as well as on adipose specific miRNA regulation and exosomal extracellular vesicle trafficking.

Since we have an ongoing interest in the role of polyunsaturated fatty acids (PUFAs) in health and disease, the article by Dr Undurti Das et al. is of great interest to us. Earlier studies from this group demonstrated that low molecular weight lipid molecules, such as linoleic acid, alpha-linolenic acid, gamma linolenic acid (GLA), dihomo-GLA, arachidonic acid (AA), eicosapentaenoic acid (EPA), and docosahexaenoic acid (DHA), have a potent antidiabetic effect, especially against drug-induced diabetes in animal models. According to these authors, AA was the most potent inhibitor of drug-induced hyperglycemia. In this study, the authors demonstrated that Protectin DX (PDX), a metabolite of DHA, which is an anti-inflammatory molecule, effectively prevented Streptozotocin-induced type-1 and type-2 diabetes in Swiss albino mice. The authors speculate that PDX is more effective in restoring β-cell dysfunction and attribute the functional restoration of these cells to anti-inflammatory action and inflammation resolution.

These studies remind me of an earlier study we conducted at the University of Minnesota in the early 1980s. In our Thrombosis Research laboratory, at that time, we were looking into the mechanisms of platelet hyperfunction in diabetic subjects. We used a rat model to study the altered prostaglandin pathways in Streptozotocin-induced type-2 diabetes (4). Using radiolabeled arachidonic acid, we followed the conversion of the AA substrate to stable metabolites of Thromboxane (TXA_2_) and Prostacyclin (PGI_2_).

(Courtesy; Dr Jonathan Gerrard, University of Minnesota)

As shown in the schematics in **Figure 1**, diabetic rat platelets produced more potent aggregatory molecule (Thromboxane) compared to the control. Vessel wall tissues produced less vasodilatory, anti-aggregatory molecule (Prostacyclin) compared to normal controls, thus promoting a pro-thrombotic state. Furthermore, islet cell transplantation in these diabetic rats normalized arachidonic acid metabolism (data not shown) in platelets as well as in vascular tissues, suggesting the vital role of pancreatic islet cells in restoring normal homeostasis.

**Figure 1:**
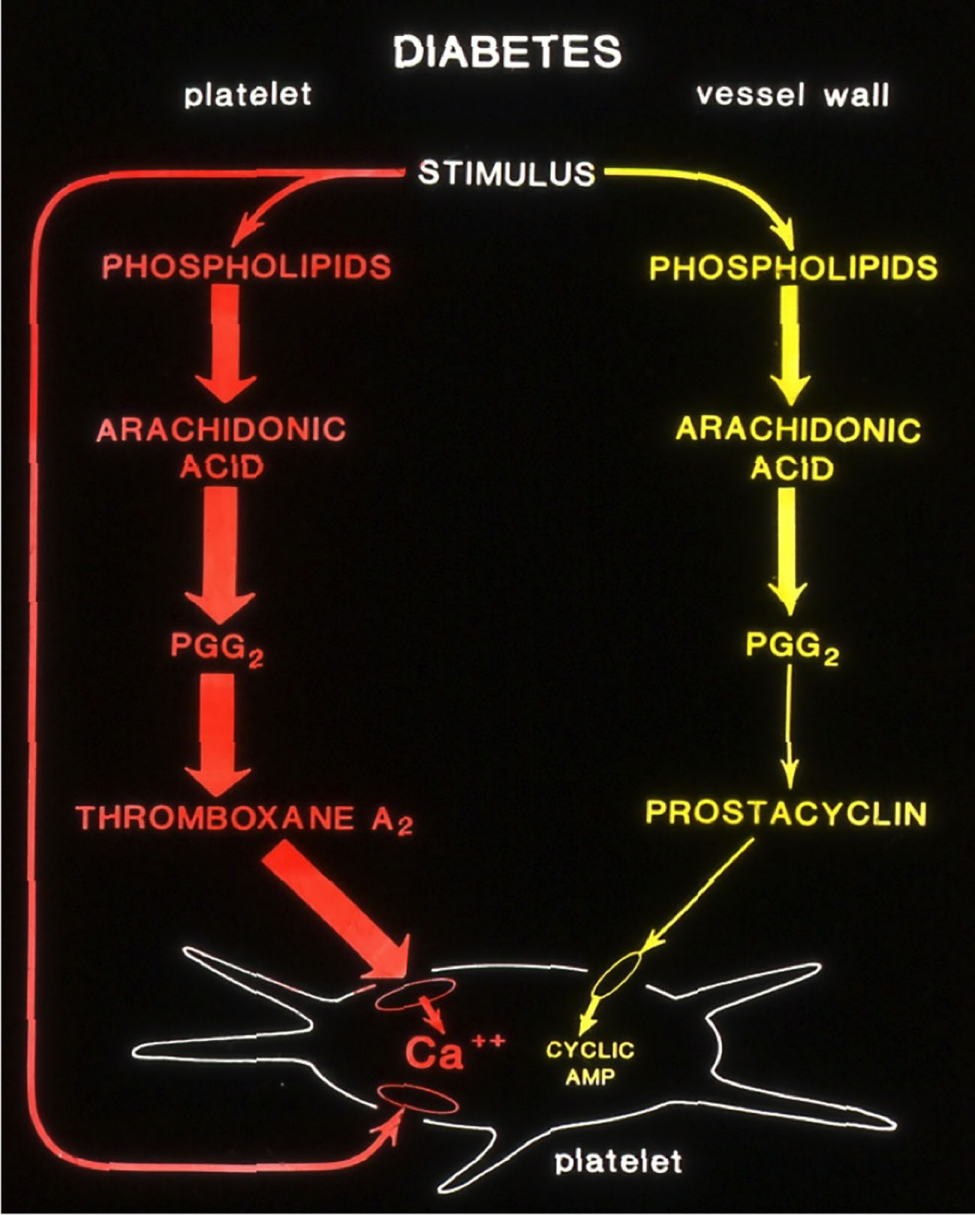
Altered production of Arachidonic acid into prostanoids in diabetic rats. (Courtesy; Dr Jonathan Gerrard, University of Minnesota).

We have just made a beginning in understanding the cellular and molecular mechanisms underlying altered glucose metabolism in diabetics. I hope publishers will put together such Research Topics on cardiometabolic diseases, hypertension, obesity, diabetes, and CVDs, as well ‘insights’ into related events such as oxidative stress, inflammation, hypertension, obesity, endothelial dysfunction, atherosclerosis, vascular diseases, and acute vascular events.

## Author contributions

The author confirms being the sole contributor of this work and has approved it for publication.
